# A Case For a Study Quality Appraisal in Survey Studies in Psychology

**DOI:** 10.3389/fpsyg.2018.02788

**Published:** 2019-01-24

**Authors:** Cleo Protogerou, Martin S. Hagger

**Affiliations:** ^1^Department of Psychology, University of Cape Town, Cape Town, South Africa; ^2^Health Psychology and Behavioral Medicine Research Group, School of Psychology, Faculty of Health Sciences, Curtin University, Perth, WA, Australia; ^3^Faculty of Sport and Health Sciences, University of Jyväskylä, Jyväskylä, Finland

**Keywords:** study quality appraisal, psychology, correlational studies, survey studies, evidence syntheses, transparency

## Introduction

The lack of replication of key effects in psychology has highlighted some fundamental problems with reporting of research findings and methods used (Asendorpf et al., 2013; Open Science Collaboration, 2015). Problems with replication have been attributed to sources of bias such as questionable research practices like HARK-ing (Kerr, 1998) or *p*-hacking (Simmons et al., 2011). Another potential source of bias is lack of precision in the conduct and methods used in psychological research, which likely introduces systematic error into data collected with the potential to affect results. A related issue is lack of accuracy in reporting study methods and findings. There is, therefore, increased recognition in the importance of transparency when reporting study outcomes to enable the scientific community to make fair, unbiased appraisals of the implications and worthiness of study findings. Lack of transparency hinders scientific progress as it may lead to erroneous conclusions regarding the implications of research findings, and may impede comparison and synthesis of findings across studies. As a result, researchers have become interested in research quality and the need for comprehensive, transparent reporting of findings (Asendorpf et al., 2013). This has resulted in calls for appropriate reporting standards and means to assess study quality (Cooper, 2011; Greenhalgh and Brown, 2017). In the present article we review the issue of study quality in psychology, and argue for valid and reliable means to assess study quality in psychology. Specifically, we contend that appropriate assessment checklists be developed for survey studies, given the prominence of surveys as a research method in the field.

## Importance of Assessing Study Quality

Study quality is the degree to which researchers conducting the study have taken appropriate steps to maximize the validity of, and, minimize bias in, their findings (Khan et al., 2011). Studies of lower quality are more likely to have limitations and deficits which introduce error variance to data that can bias results and their interpretation. Studies of higher quality are less likely to include these errors, or more likely to provide clear and transparent reporting of errors and limitations, resulting in greater precision and validity of findings and their interpretation (Oxman and Guyatt, 1991; Moher et al., 1998). Study quality assessment came to prominence from the evidence-based medicine approach, which focussed on identifying, appraising, and synthesizing medical research (Guyatt et al., 1992). The ideas have since been applied to other disciplines, including the behavioral and social sciences (Michie et al., 2005; APA, 2006b). Assessment of study quality has several advantages, such as identifying the strengths and weaknesses in evidence, providing recommendations for interventions, policy, and practice, and improving research and publication standards (Greenhalgh, 2014; Greenhalgh and Brown, 2017). Moreover, in the context of evidence syntheses, study quality can be used to screen studies for inclusion, identify sources of bias in the results, and measure the impact of study quality on the results through subgroup and sensitivity analyses (Johnson et al., 2014).

Study quality assessment is typically performed with the use of a checklist or “tool,” containing a series of quality-related items. Recent reviews have identified a large number of tools (*N* = 193) used to assess study quality in the health and social sciences (Katrak et al., 2004). Tools have been adopted to appraise the quality of studies with specific designs such as experimental (e.g., Jadad et al., 1996), systematic reviews and meta-analyses (e.g., Oxman and Guyatt, 1991), and qualitative (e.g., Long and Godfrey, 2004) research. Generic tools, purported to be applicable to multiple study designs and across multiple disciplines, also exist (e.g., Glynn, 2006). However, most quality assessment tools have not been developed with sufficient attention to validity and reliability (Katrak et al., 2004; Moyer and Finney, 2005; Crowe and Sheppard, 2011; Johnson et al., 2014), and no quality assessment tool has been universally endorsed as fully sufficient to assess study quality (Alderson et al., 2003). Prominent criticisms of existing tools refer to the absence of validity and reliability checks in their development, as well as the absence of clear guidance on assessment procedures and scoring (Moyer and Finney, 2005; Crowe and Sheppard, 2011). Despite these limitations, quality assessment tools have been applied extensively across health and social sciences, especially in evidence syntheses.

In psychology, study quality assessment was not recognized as an integral component of the research process until relatively recently. Formal recommendations for conducting quality appraisal in meta-analyses in psychology initially appeared in the Meta-Analysis Reporting Standards (MARS) and the American Psychological Association publication manual (APA, 2006a; Appelbaum et al., 2018). Since the publication of these guidelines, awareness and application of quality appraisal has expanded rapidly, and, while still not fully accepted as standard practice, quality appraisal is frequently viewed as an essential component of evidence syntheses in psychology.

## Quality Assessment in Psychology Survey Research

Many studies in psychology adopt survey methods. Surveys are used extensively across psychology disciplines to examine relations among psychological constructs measured through psychometric scaling, and to test hypotheses with respect to relations among constructs (Check and Schutt, 2012; Ponto, 2015). However, despite the increasing demand for quality appraisal and the pervasiveness of survey designs in psychology, there are no quality assessment tools developed specifically for survey research in psychology. Given the centrality of survey methods (Ponto, 2015), development of a dedicated, fit-for-purpose quality tool should be considered a priority.

The lack of tools to appraise study quality in survey research has led researchers to adapt tools from other disciplines, or to identify relevant quality criteria from scratch and develop their own tool. To illustrate, in their meta-analysis linking job satisfaction to health outcomes, Faragher et al. (2005) stated that “…a thorough search failed to identify criteria suitable for correlational studies. A measure of methodological rigor was thus developed specifically for this meta-analysis” (p. 107). More recently, Hoffmann et al. (2017) in a meta-analysis of cognitive mechanisms and travel mode choice stated: “No suitable quality assessment tool was found to assess such survey studies. We therefore applied three criteria that were highlighted across six previous studies recommending bias assessment in correlational studies” (p. 635). In the absence of quality appraisal tools, some meta-analyses, especially those including intervention studies, have implemented universal reporting guidelines as proxies for study quality appraisal (Begg et al., 1996; Jarlais et al., 2004; Von Elm et al., 2007; Moher et al., 2009). Although these universal reporting guidelines are well-accepted, they are not, strictly speaking, quality appraisal tools, and it is unclear if they are suitable for assessing study quality in psychology, including research adopting survey methods.

The application of different tools, or individual criteria, to assess research quality, has a number of drawbacks. First, applying different tools to the same body of evidence can produce different conclusions about the quality of the evidence. This would have serious implications within the context of a meta-analysis, as the effect size may vary as a function of the quality appraisal tool used. For example, Armijo-Olivo et al. (2012) compared the performance of two frequently-used quality appraisal tools, the Cochrane Collaboration Risk of Bias Tool (CCRBT; Higgins and Altman, 2008) and the Effective Public Health Practice Project Quality Assessment Tool (EPHPP; Jackson and Waters, 2005) in a systematic review of the effectiveness of knowledge translation interventions to improve the management of cancer pain, and found that both tools performed differently. Similarly, Jüni et al. (1999) applied 25 quality appraisal scales to the results of a meta-analysis comparing low-molecular-weight heparin with standard heparin for clot prevention in general surgery, and found that different quality scales produced different conclusions regarding the relative benefits of heparin treatments. For studies classed as high quality on some tools, there was little difference in outcome for two types of heparin, whereas for studies classed as high quality on others, one was found to be superior. Moreover, the overall effect size was positively associated with scores on some quality tools but inversely associated with scores on others. Second, the adapted quality assessment tools used by psychologists were not developed to evaluate research in psychology, and may consequently lack validity, and incompletely cover important study quality components.

## Problems Arising from Quality Assessment Methods: An Illustration

To illustrate the longstanding problems resulting from the absence of a fit-for-purpose tool and the application of a variety of quality appraisal strategies, we provide examples from a brief summary of quality assessments from meta-analyses of psychological survey research (Table 1)^1^ We identified two prominent limitations of the tools: the quality criteria adopted and the scoring strategies employed.

**Table 1:** Summary of quality assessment tool characteristics in studies reviewed.

**Study**	**Quality tool used**	**Discipline**	**Number of quality criteria**	**Scoring Strategy**	**Type of scoring**	**Guide or explanation of criteria provided?**	**Quality classification system**
Cuijpers et al., 2010	Developed quality criteria from a review of empirically supported psychotherapies (Chambless and Hollon, 1998) and from methodological quality recommendations of the Cochrane Collaboration (Higgins and Green, 2006)	Clinical/counseling psychology	8	Checks of whether quality criteria were met	A sum of criteria met by the study	Explanation of criteria provided by authors	A study that met all quality criteria was classified as high quality, otherwise it was classified as lower quality
Faragher et al., 2005	Developed quality criteria based on guidelines on research procedures in organizational psychology and expert consensus	Organizational/ industrial/ occupational psychology.	10	Each criterion was given a 0 score (rating) for unacceptable rigor or 1 for acceptable rigor	A summated rigor score computed (range 0–10)	Not indicated	A study that met all 10 criteria was classified as of acceptable rigor, otherwise it was classed as of unacceptable rigor
Godfrey et al., 2015	Effective Public Health Practice Project Quality Assessment Tool (EPHPP; Jackson and Waters, 2005)	Clinical/counseling psychology; health psychology; applied psychology	6	Each criterion was given 1 point for a weak quality rating, 2 points for a moderate quality rating, and 3 points for a strong quality rating	Sum of scores divided by total number of applicable criteria	Tool is published with guide	Studies of weak quality had a rating of 3, while studies of moderate quality had a rating of 2, and studies of strong quality had a rating of 1.
Hagger et al., 2017	Quality criteria adapted from the National Institutes of Health Quality Assessment Tool for Observational Cohort and Cross-Sectional Studies (National Institutes of Health, 2014), and from other quality checklists used in cross-sectional survey designs (Jack et al., 2010; Husebø et al., 2013; Oluka et al., 2014).	Health psychology; social psychology	16	A score of 1 was assigned for each criterion met and a score of zero 0 for each criterion not met or when there was insufficient information provided to evaluate the criterion	Three types of scoring: weighted checklist score out of 10; Tertile division of checklist scores; Average checklist score	Explanation of criteria provided by authors	Tertile division of scores on the quality checklist resulted in studies above the upper tertile classified as high quality and studies below the lower tertile classified as low quality. Also, studies scoring an average of ≥6 were classified as high quality and studies scoring an average score of < 6 were classified as low quality
Hoffmann et al., 2017	Criteria for correlational designs recommended in six previous studies (Gauthier, 2003, (Effective Public Health Practice Project [EPHPP], Jackson and Waters, 2005; Von Elm et al., 2007; Wong et al., 2008; Pace et al., 2012; National Heart, Lung, and Blood Institute, 2014)	Applied psychology; traffic psychology	5	A score of one (1) assigned for criteria met and a score of zero (0) assigned for criteria not met or with insufficient information provided.	Total mean score	Explanation of criteria provided by authors	Studies that received an overall score > 2 were rated as high quality, those receiving scores 1–2 were rated as medium quality, and those receiving a < 1 score were rated low quality
Pantelic et al., 2015	Adapted version of the Cambridge Quality Checklists (CQC; Murray et al., 2009)	Cultural psychology; health psychology	8	Each criterion was assigned a numerical score between 0 and 6	One hundred per cent score would indicate the maximum possible score across all correlations in a study	Tool is published with guide	Manuscript reported quality scores but did not formally classify studies according to quality
Protogerou et al., 2018	Adapted version of a generic quality appraisal tool Glynn, 2006	Health psychology; social psychology; applied psychology	23	Each quality criterion was checked as being present (yes = 1); absent (no = 2); unclear (3) or not applicable (4)	A ratio of the “yes” answers by the total applicable items, multiplied by 100	Tool is published with guide	In line with the tool's guidelines, studies receiving a total score of < 75% were classified as of questionable quality, whereas studies with a total score of ≥75% were classified of acceptable quality.
Quon and Mcgrath, 2014	Eight criteria to assess study quality	Health psychology	8	Not indicated in manuscript.	Not indicated	Not indicated in manuscript	High quality or low quality (cut-offs not indicated)
Santos et al., 2017	A short, adapted version of the Joanna Brigs Institute critical appraisal checklist for studies reporting prevalence data (Joanna Briggs Institute, 2014)	Health psychology; sports psychology.	5	Each quality criterion was scored as yes, no, unclear or not applicable No corresponds to a limitation in the respective methodological category	The tool does not allow for numerical summative scoring Quality was used in sensitivity analysis implying summative scoring but no details provided	Tool is published with guide	Not clearly indicated
Young et al., 2014	Checklist informed by the Strengthening of Reporting of Observational Studies in Epidemiology (STROBE: Von Elm et al., 2007) and Consolidated Standards for Reporting Trials (CONSORT: Moher et al., 2010) statements, augmented with items from two reviews (Rhodes et al., 2009; Plotnikoff et al., 2013); and a list of “strong model characteristics” (Noar and Zimmerman, 2005)	Health psychology; sports psychology	11	Each quality criterion was scored as present (Y), absent (N), unclear or inadequately described' (0) or not applicable (n/a)	Sum of scores of present quality criteria	Explanation of criteria provided by authors	Not clearly indicated

### Quality Criteria

The number of assessed quality criteria ranged between 5 and 23 across the meta-analyses. Also, the type and origin of quality criteria was highly variable. For instance, two meta-analyses (Faragher et al., 2005; Cuijpers et al., 2010) developed quality criteria specifically for their research, while seven meta-analyses (Young et al., 2014; Godfrey et al., 2015; Pantelic et al., 2015; Hagger et al., 2017; Hoffmann et al., 2017; Santos et al., 2017) applied adapted criteria from existing quality tools, reporting guidelines, and literature searches. One study indicated quality criteria without explaining how those were developed or chosen (Quon and Mcgrath, 2014). Although most studies appraised sampling and recruitment procedures, there was variability in the criteria adopted. For example, Hoffmann et al. (2017) appraised whether or not the sample size was sufficient to analyze data using structural equation modeling, while (Quon and Mcgrath, 2014) adopted an absolute total sample size (*N* = 1000) as their criterion for quality. Similarly, most studies assessed the “appropriateness” of statistical analyses, without clarifying what was considered “appropriate”.

### Assessment and Scoring

There was substantive variability in the scoring strategies used to assess study quality across the meta-analyses. Some meta-analyses adopted numerical scoring systems calculating overall percentages, summary scores, and mean scores for the quality criteria adopted (e.g., Protogerou et al., 2018), while other studies did not employ numerical or overall scoring (e.g., Santos et al., 2017). In relation to this, most studies classified studies in terms of high (or “acceptable”) quality vs. low (or “questionable”) quality, while others did not categorize studies in terms of quality. Some studies indicated that quality assessment was informed by published manuals or guidelines on quality criteria, while other studies provided no information on the guidelines or definitions of criteria adopted.

Given the disparate quality appraisal strategies adopted by the meta-analyses, we contend, in line with Armijo-Olivo et al. (2012) and Jüni et al. (1999), that quality assessment outcomes are dependent on the specific tool applied, and that different tools might lead to different conclusions on quality. Moreover, it would be difficult to replicate the quality assessment procedures adopted in most of these meta-analyses, given the limited information provided. We also note that quality criteria relevant to psychological survey studies were missed in the quality assessment on some meta-analyses. For example, ethical requirements, such as consent and debriefing procedures, and response and attrition rates were not checked consistently.

## Conclusion and Recommendations

Assessment of study quality is an important practice to promote greater precision, transparency, and evaluation of research in psychology. Assessing the quality of studies may permit researchers to draw effective conclusions and broader inferences with respect to results from primary studies, and when synthesizing research across studies, provide the opportunity to evaluate the general quality of research in a particular area. Given the prominence of survey research in psychology, the development of appropriate means to assess the quality of survey research would yield considerable benefits to researchers conducting, and data analysts evaluating, survey research. We argue that a fit-for-purpose quality appraisal tool for survey studies in psychology is needed. We would expect the development of such a tool to be guided by discipline-specific research standards and recommendations (BPS, 2004; APA, 2006b; Asendorpf et al., 2013). We would also expect the tool to be developed through established methods, such as expert consensus, to ensure satisfactory validity and reliability of the resulting tool (for examples and discussion of these strategies see Jones and Hunter, 1995; Jadad et al., 1996; Crowe and Sheppard, 2011; Jarde et al., 2013; Waggoner et al., 2016).

## Author Contributions

CP and MH conceived the ideas presented in the manuscript and drafted the manuscript.

### Conflict of Interest Statement

The authors declare that the research was conducted in the absence of any commercial or financial relationships that could be construed as a potential conflict of interest.

## Footnotes

^1^A comprehensive version of Table 1 with full details of study quality criteria is provided online: https://osf.io/wbj5z/?view_only=ffbb265cf43f498999ab69bc57c60eb5
